# YOLOv10-kiwi: a YOLOv10-based lightweight kiwifruit detection model in trellised orchards

**DOI:** 10.3389/fpls.2025.1616165

**Published:** 2025-08-25

**Authors:** Jie Ren, Wendong Wang, Yuan Tian, Jinrong He

**Affiliations:** College of Mathematics and Computer Science, Yan’an University, Yan’an, Shaanxi, China

**Keywords:** kiwifruit detection, YOLOv10, lightweight network, HetConv, CCFM, MPDIou

## Abstract

To address the challenge of real-time kiwifruit detection in trellised orchards, this paper proposes YOLOv10-Kiwi, a lightweight detection model optimized for resource-constrained devices. First, a more compact network is developed by adjusting the scaling factors of the YOLOv10n architecture. Second, to further reduce model complexity, a novel C2fDualHet module is proposed by integrating two consecutive Heterogeneous Kernel Convolution (HetConv) layers as a replacement for the traditional Bottleneck structure. This replacement enables parallel processing and enhances feature extraction efficiency. By combining heterogeneous kernels in sequence, C2fDualHet captures both local and global features while significantly lowering parameter count and computational cost. To mitigate potential accuracy loss due to lightweighting, a Cross-Channel Fusion Module (CCFM) is introduced in the neck network. This module incorporates four additional convolutional layers to adjust channel dimensions and strengthen cross-channel information flow, thereby enhancing multi-scale feature integration. In addition, a MPDIoU loss function is introduced to overcome the limitations of the traditional CIoU in terms of aspect ratio mismatch and bounding box regression, accelerating convergence and improving detection accuracy. Experimental results demonstrate that YOLOv10-Kiwi achieves a model size of only 2.02 MB, with 0.51M parameters and 2.1 GFLOPs, representing reductions of 80.34%, 81.11%, and 68.18%, respectively, compared to the YOLOv10n baseline. On a self-constructed kiwifruit dataset, the model achieves 93.6% mAP@50 and an inference speed of 74 FPS. ​​YOLOv10-Kiwi offers an efficient solution for automated kiwifruit detection on low-power agricultural robots.

## 1 Introduction

Kiwifruit is widely welcomed for its abundance of vitamin C, dietary fiber, and a variety of trace elements (Richardson et al., 2018). Meixian County in Shaanxi Province, as the core kiwi-producing region in China, has seen its industry not only boost the local economy and rural revitalization but also gain a significant position in the global market. Modern T-shape cultivation enhances orchard management, but harvesting remains labor-intensive, facing labor shortages and high physical demands (Deng et al., 2023). Automation technologies can streamline harvesting processes, improving efficiency and reducing labor dependency (Fu et al., 2024).

Traditional kiwifruit detection methods mainly rely on color features, morphological features, and machine vision techniques, using handcrafted features for fruit segmentation and identification. For example (Scarfe, 2012), combined RGB color subtraction with Sobel filters to detect kiwis and their calyx edges, using template matching for identification, but did not consider fruit shape features (Zhan et al., 2013) used the Adaboost algorithm to optimize kiwifruit segmentation and localization in field conditions. By analyzing RGB, HSV, and Lab color spaces, they selected the most discriminative channels between fruit and background to improve detection accuracy. With the rising demand for nighttime automated harvesting (Longsheng et al., 2015), proposed a machine vision method based on artificial lighting to overcome the impact of natural light variability, thereby enhancing detection stability. Overall, these traditional methods are significantly affected by lighting conditions and fruit morphology variations, resulting in limited generalization ability and poor adaptability to complex environments. Consequently, researchers have begun exploring deep learning-based approaches to overcome these limitations.

Current deep learning-based kiwi fruit detection algorithms can be broadly categorized into two-stage and one-stage detection methods. Two-stage methods, such as Faster R-CNN (Faster r-cnn: Towards real-time object detection with region proposal networks, 2015), Cascade R-CNN (Cai and Vasconcelos, 2018), and Mask R-CNN (He et al., 2017), first generate region proposals followed by classification and bounding box refinement. These models achieve high detection accuracy and robustness but are computationally intensive and slower. In contrast, one-stage detectors—such as the YOLO series (Jiang et al., 2022), SSD (Liu et al., 2016), and EfficientDet (Tan et al., 2020)—offer faster inference and lower computational cost, gradually becoming the mainstream for real-time detection. For instance (Suo et al., 2021)used YOLOv4 for multi-class kiwifruit detection, achieving a maximum mAP of 91.9% with a per-image inference time of 25.5 ms, reducing robotic misoperations (Yao et al., 2021) improved YOLOv5 by adding a small object detection layer, introducing a SE attention mechanism, and optimizing the loss function to accurately detect kiwi defects (Xia et al., 2022) integrated an attention module into YOLOv7 and employed spectral techniques to improve detection precision.

With the increasing demand for lightweight models, researchers have proposed various optimizations to the YOLO series to reduce computational load while maintaining accuracy. In the fruit detection domain, numerous efficient improvements have been made. For example (Liu et al., 2024) proposed the Faster-YOLO-AP model, which used structural simplification and lightweight convolutions to compress the parameter count to 0.66M and FLOPs to 2.29G, while retaining high apple detection accuracy (Zhao et al., 2024). introduced YOLO-Granada, which maintained accuracy close to YOLOv5s while reducing parameters, computation, and model size to 54.7%, 51.3%, and 56.3% of the original, respectively, greatly enhancing pomegranate detection efficiency. In the context of kiwifruit detection, several lightweight models have also shown promising results. For example (Fu et al., 2021) optimized YOLOv3-tiny to develop the DY3TNet model, improving detection accuracy with 3×3 and 1×1 convolutions in layers 5 and 6, and compressing weights to 27 MB with an inference time of just 34 ms per image (Gao et al., 2022) used GhostNet to replace the original CSP-Darknet53 backbone and partially substituted standard convolutional layers, significantly reducing computational load (Zhou et al., 2022). proposed an enhanced YOLOX-S model, reducing parameters by 44.8% and increasing detection speed by 63.9% through feature map simplification, activation function optimization, and loss function improvements.

Despite these advancements in model lightweighting and detection performance, challenges remain in kiwifruit detection tasks. Existing models still face high parameter counts and computational burdens. While complex feature fusion and convolution operations improve accuracy, they exacerbate computational bottlenecks, limiting deployment and real-time application on edge devices. To address this, this paper proposes a lightweight kiwifruit detection model based on YOLOv10 (Wang et al., 2024), aiming to reduce computational complexity while maintaining high detection accuracy. The main contributions are as follows:

1. Construction of a diversified kiwifruit dataset. A total of 1,280 images were collected from real orchards under various angles and scenes, covering complex lighting conditions, occlusions, and diverse morphological appearances of the fruit.
2. Proposal of the lightweight YOLOv10-Kiwi model. Based on YOLOv10n, the network is compressed via scaling factor adjustments. The lightweight C2fDualHet module is designed for feature extraction, the CCFM structure is introduced to enhance feature fusion, and the MPDIoU loss function is adopted to improve bounding box regression accuracy.
3. Validation through comparative and ablation experiments. The proposed model’s effectiveness is demonstrated by extensive experiments against mainstream detection methods.

## 2 Materials and methods

### 2.1 Dataset construction

#### 2.1.1 Data acquisition

**Figure 1:**
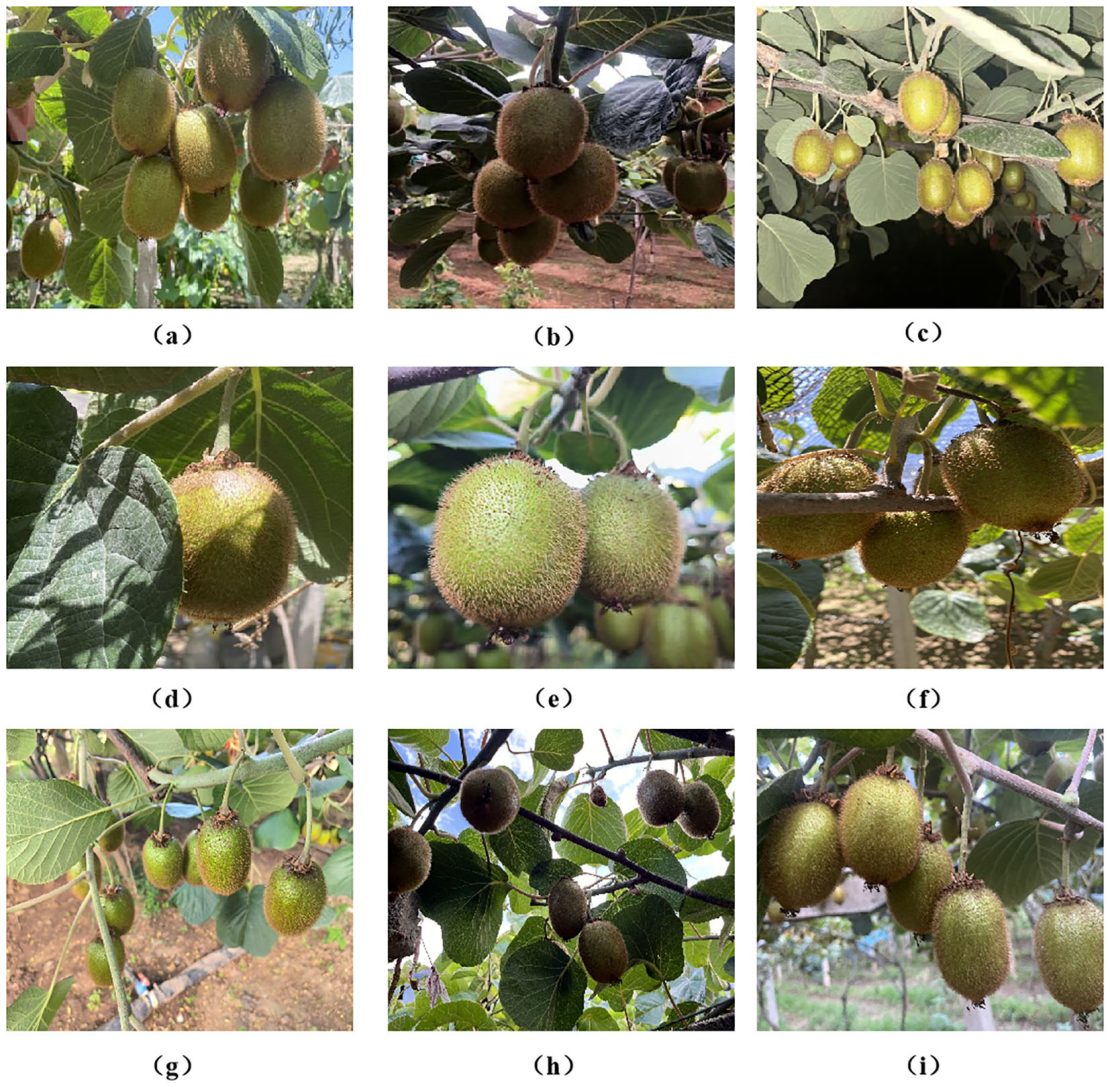
Images of kiwifruit under different conditions: **(a)** Sunny day, **(b)** Cloudy day, **(c)** Night, **(d)** Leaf obstruction, **(e)** Fruit overlap, **(f)** Branch obstruction, **(g)** Top-down shot, **(h)** Low-angle shot, and **(i)** Eye-level shot.

The image dataset used in this study was collected from July to August 2024 in a commercial kiwifruit orchard in Meixian County, Shaanxi Province. Xuxiang kiwifruit images grown under trellis conditions were captured using iPhone 13 and 14 devices at various angles (top-down, oblique, upward) and lighting conditions (front lighting, backlighting, flash at night). Sampling was conducted on sunny and cloudy days, as well as at night (with flash), to simulate realistic harvesting scenarios. Rainy conditions were excluded since harvesting typically does not occur during such weather. This summer sampling phase coincided with the fruit maturation period, ensuring consistency in fruit size and color—crucial for effective model training in robotic applications. A total of 1,280 images were collected: 828 on sunny days (411 in the morning, 417 in the afternoon), 190 under cloudy skies, and 262 at night. All images were resized to 640 × 640 pixels and reflect challenging real-world conditions, including dense occlusion, uneven lighting, clustered fruit, and complex backgrounds. Representative samples are shown in **Figure 1**, which illustrates these visual challenges more intuitively. This figure highlights the diversity and complexity of orchard environments, underscoring the need for robust detection under variable field conditions.

#### 2.1.2 Data annotation

**Figure 2:**
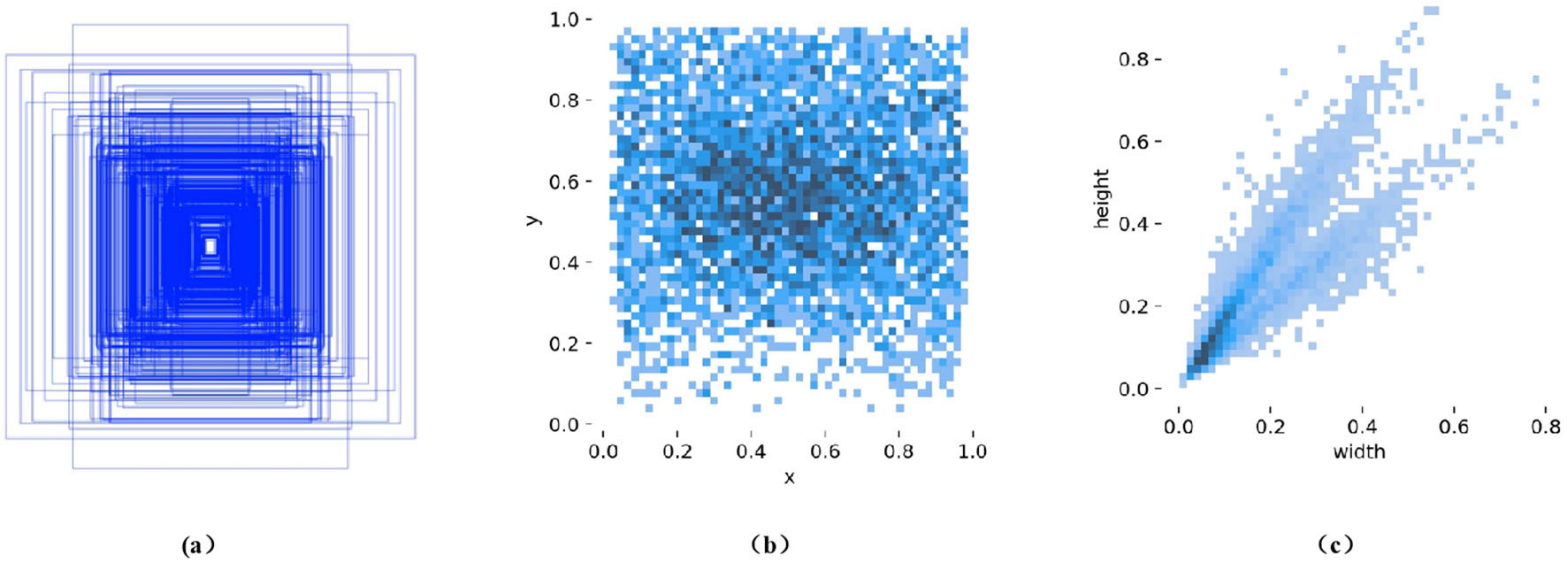
Distribution of bounding box sizes and label scales in the kiwifruit dataset: **(a)** Boundary Box Size Distribution, **(b)** Distribution of object center points (color intensity represents density), and **(c)** Scatter plot of width-to-height ratios.

In this experiment, we used LabelImg software to annotate kiwi fruit images with rectangular bounding boxes, assigning a single category label “kiwi.” After the annotation process was completed, the data was saved in YOLO format as.txt files, with each line containing the class label, the normalized center coordinates (x_center, y_center), width, and height—scaled to the [0, 1] range. The dataset was divided into training, validation, and test sets in a ratio of 8:1:1. Specifically, the 1,280 images were distributed as follows: 772 images in the training set (containing 3,318 instances), 253 images in the validation set (1,010 instances), and 255 images in the test set (1,022 instances). The statistical analysis results, as shown in **Figure 2**, indicate variations in both the scale and distribution of the annotated bounding boxes, which are consistent with the natural growth characteristics of kiwi fruits. **Figure 2a** illustrates the distribution of width-to-height ratios of the bounding boxes, reflecting significant differences in the sizes of the fruits. **Figure 2b** displays the distribution of object center points, with color intensity representing density, showing that most objects are concentrated near the center of the images. **Figure 2c** presents a scatter plot of width-to-height ratios, which tend to cluster around 1, indicating that the bounding boxes are regular in shape, with similar width and height. These characteristics provide valuable data support for subsequent algorithm optimization.

### 2.2 Novel network construction

YOLOv10 serves as the foundation for this study, offering a well-balanced trade-off between detection accuracy and inference speed through its lightweight classification head, spatial-channel decoupled downsampling, and rank-based architectural simplifications. These structural advantages make it suitable for real-time detection tasks. However, when applied directly to kiwifruit detection in trellised orchard environments, the default YOLOv10n model reveals several limitations. Its computational load and memory usage remain relatively high for resource-constrained edge devices, and the backbone structure lacks specialized design for lightweight feature extraction under conditions such as dense occlusion, variable lighting, and scale variation. In addition, the neck network provides limited cross-channel fusion, which weakens its ability to capture fine-grained multi-scale features, especially for small or overlapping fruits. Furthermore, the use of the CIoU loss function in bounding box regression often results in suboptimal localization performance when dealing with irregular fruit shapes and aspect ratio mismatches.

**Figure 3:**
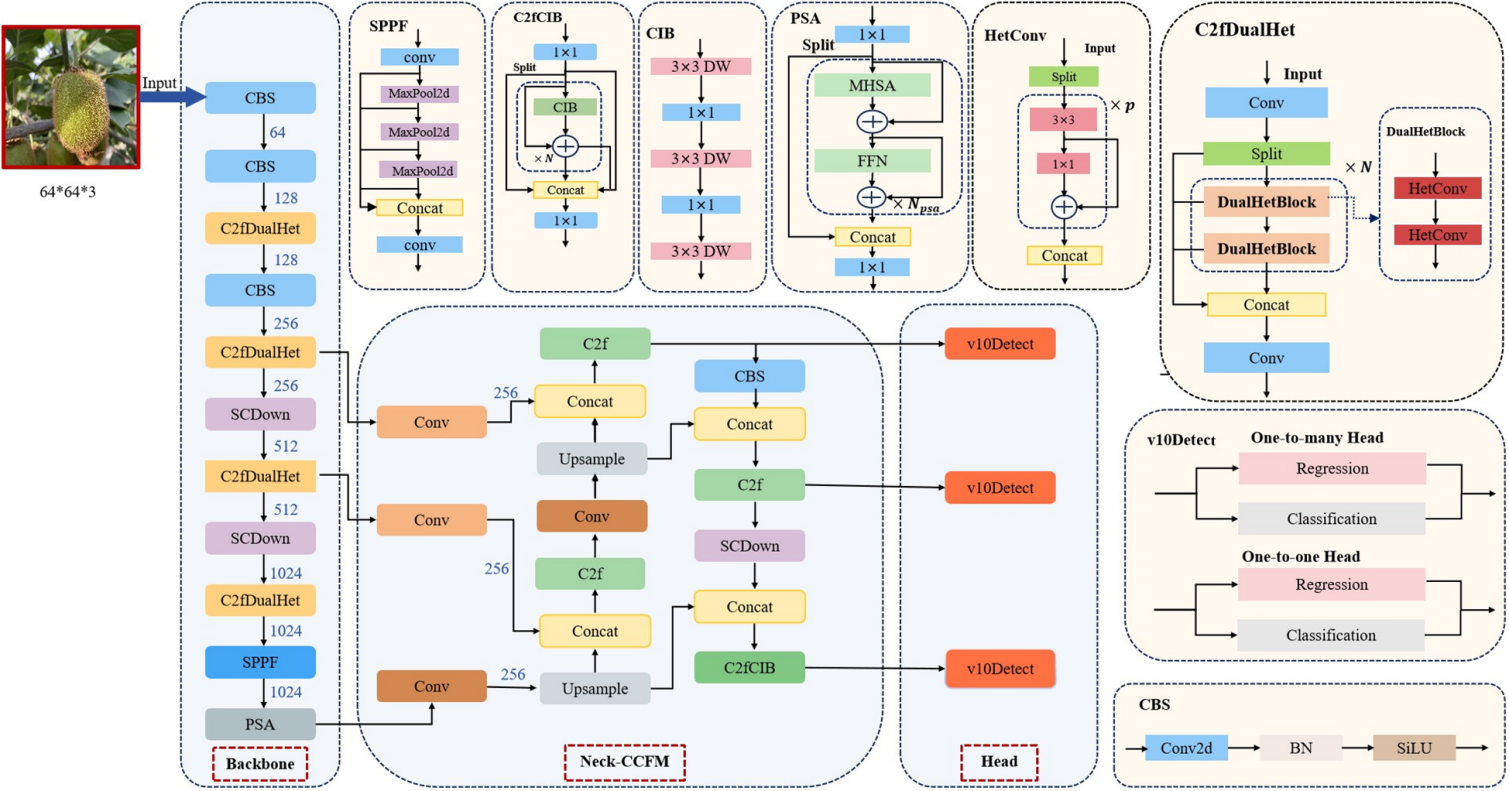
Schematic of the proposed lightweight network structure.

To address these challenges, we propose YOLOv10-Kiwi, a lightweight and efficient detection model specifically optimized for kiwifruit detection in complex orchard conditions. As illustrated in **Figure 3**, the model architecture incorporates three key structural refinements. The original C2f block in the YOLOv10n backbone is replaced with the C2fDualHet module, which uses two consecutive HetConv layers to improve feature representation while significantly reducing the number of parameters and computational cost. The neck is enhanced with a Cross-Channel Fusion Module (CCFM), which strengthens the integration of multi-scale and inter-channel information. Four additional convolutional layers are incorporated to refine channel dimensions and boost inter-channel interactions, thereby improving detection robustness in the presence of occlusion and size variability. Lastly, the head adopts a decoupled structure for classification and regression, with the conventional CIoU loss replaced by the Minimum Point Distance IoU (MPDIoU) loss. This loss function improves localization accuracy and convergence stability, particularly in scenes with densely packed or partially visible targets. With these targeted improvements, YOLOv10-Kiwi achieves a compact and efficient design that maintains real-time performance while enhancing detection accuracy, making it well-suited for deployment on low-power edge devices in agricultural applications.

#### 2.2.1 C2fDualHet

To further reduce the number of model parameters, this study integrates HetConv (Heterogeneous Kernel Convolution) (Singh et al., 2019) into the YOLOv10 framework. HetConv combines two kernel sizes—3×3 and 1×1—and introduces a configurable parameter *P* to control the proportion of channels processed by each type, achieving an effective balance between spatial representation and computational efficiency.

**Figure 4:**
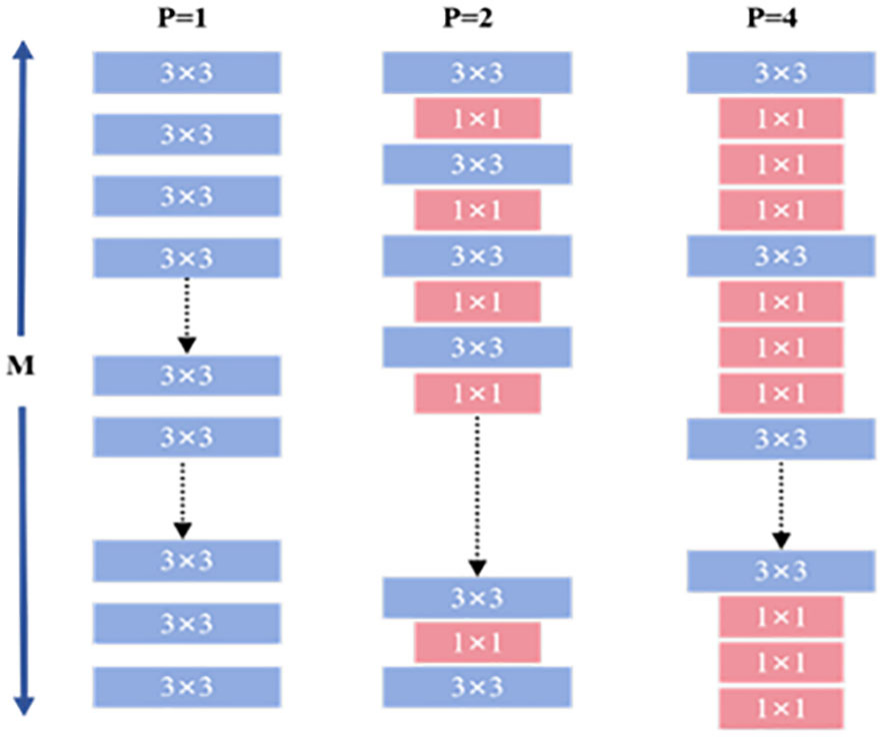
HetConv convolutional structure.


As shown in **Figure 4**, the input channels M are divided into P parts: M/P channels are processed with 3×3 convolution kernels, while the remaining channels use 1×1 kernels. In a standard convolution operation, assume the input feature map has dimensions $D_{i}\times D_{i}\times M$ , where $D_{i}$ is the width and height of the input feature map, and M is the number of input channels. The output feature map has dimensions $D_{o}\times D_{o}\times N$ , where $D_{o}$ is the width and height of the output feature map, and N is the number of output channels. The output feature map is obtained using N convolution kernels, each of size K×K×M , where K is the kernel size. Based on this, the computational cost of this layer can be expressed as:

(1) $$\begin{array}{c} FL_{s}=D_{0}\times D_{0}\times M\times N\times K\times K \end{array}$$

**Figure 5:**
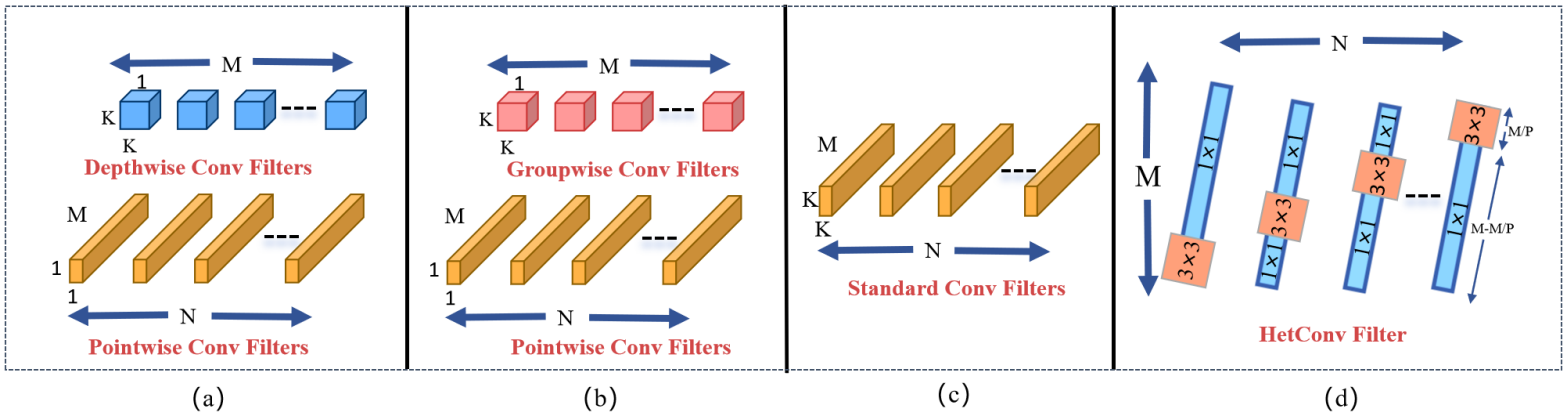
Comparison between HetConv and other efficient convolutional filters: **(a)** Standard convolution, **(b)** Depthwise convolution, **(c)** Pointwise convolution, **(d)** Group convolution, and **(e)** HetConv.

As shown in Equation 1, the computational cost of a convolutional layer is influenced by several key factors: the kernel size K , the image dimensions $D_{i}\times D_{i}$ , the number of input channels M, and the number of output channels N . In traditional standard convolution, this computational cost is often quite substantial, posing challenges for computational resources. In the pursuit of more efficient convolution methods, researchers have continuously explored and developed various convolutional models. Notable among these are depthwise convolution (DWC) (Howard, 2017), pointwise convolution (PWC) (Chollet, 2017), and group convolution (GWC) (Guo et al., 2019). While these models significantly reduce computational load, they typically introduce an additional unit of latency. In contrast, HetConv not only effectively reduces computational cost but also achieves zero latency, which is especially important in applications with high real-time requirements. **Figure 5** illustrates a comparison between HetConv and other convolution filters in terms of computational efficiency and latency.

In a specific HetConv layer L, the convolution operation uses K×K kernels over P channels, with its computational cost denoted as $FL_{K}$ .


(2) $$\begin{array}{c} FL_{K}=\left(D_{0}\times D_{0}\times M\times N\times K\times K\right)/P \end{array}$$

For the remaining (M - M/P) channels, 1×1 kernels are applied, with a corresponding computational cost denoted as $FL_{1}$ .


(3) $$\begin{array}{c} FL_{1}=\left(D_{0}\times D_{0}\times N\right)\times \left(M-\frac{M}{P}\right) \end{array}$$

Therefore, the total computational cost of layer L can be expressed as the sum of $FL_{K}$ and $FL_{1}$ .


(4) $$\begin{array}{c} FL_{HC}=\;FL_{K}+FL_{1} \end{array}$$

Compared to standard convolution, the computational reduction ratio $R_{HC}$ of HetConv can be formulated accordingly.


(5) $$\begin{array}{c} R_{HC}=\frac{FL_{K}+FL_{1}}{FL_{S}}=\frac{1}{P}+\frac{1-1/P}{K^{2}} \end{array}$$

The computational formulations of Equations 2–5 provide a quantitative basis for understanding the efficiency advantages of HetConv over standard convolution, particularly in reducing FLOPs and parameter counts through mixed kernel usage. When P = 1, HetConv degenerates into standard convolution. HetConv retains K×K kernels (e.g., 3×3) only on a portion of the channels to capture critical spatial correlations, while applying 1×1 kernels on the remaining channels to reduce computational complexity. This design preserves essential spatial information while avoiding the high computational cost associated with applying large kernels across all channels. In terms of computational efficiency and parameter reduction, HetConv significantly outperforms standard convolution. The use of 1×1 kernels greatly reduces FLOPs and the number of model parameters, making the model more lightweight and vastly more efficient than standard convolution. Thus, we construct the DualHetBlock using two consecutive HetConv modules to replace the Bottleneck in the original C2f, naming it C2fDualHet.

#### 2.2.2 Cross-scale convolutional fusion module

In the complex natural environment of kiwifruit orchards, fruits are often affected by factors such as occlusion, uneven lighting, and clustered distribution. These challenges lead to significant variations in the morphology and scale of the targets, thereby reducing the accuracy and robustness of detection models. To enhance the model’s capability for multi-scale feature representation and fusion, an improved Cross-scale Convolutional Fusion Module (CCFM) (Zhao et al., 2024) is introduced, along with a redesigned feature fusion pathway within the network.

**Figure 6:**
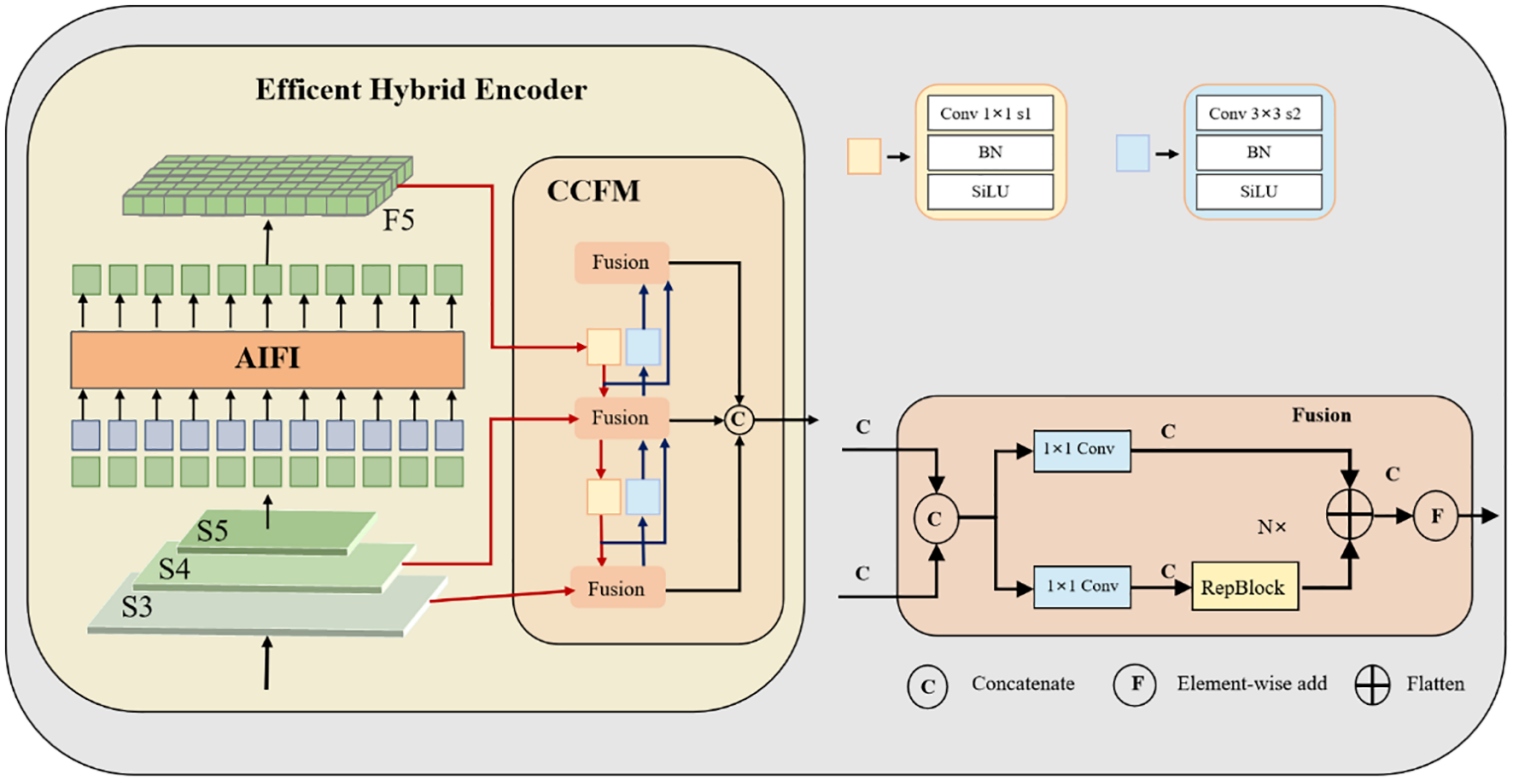
Architecture of the original cross-channel fusion module (CCFM).

As shown in **Figure 6**, this study adopts a processing strategy for the final layer of efficient hybrid encoder (F5) similar to that illustrated in the figure. Specifically, a 1×1 convolution is added after the PSA module in YOLOv10 to reduce the number of channels from 1024 to 256, thereby decreasing the parameter count while maintaining spatial dimensions and promoting inter-channel information interaction. In the diagram, shallow features S3 and S4 are compressed via 1×1 convolutions to ensure dimensional consistency before multi-scale feature fusion.

**Figure 7:**
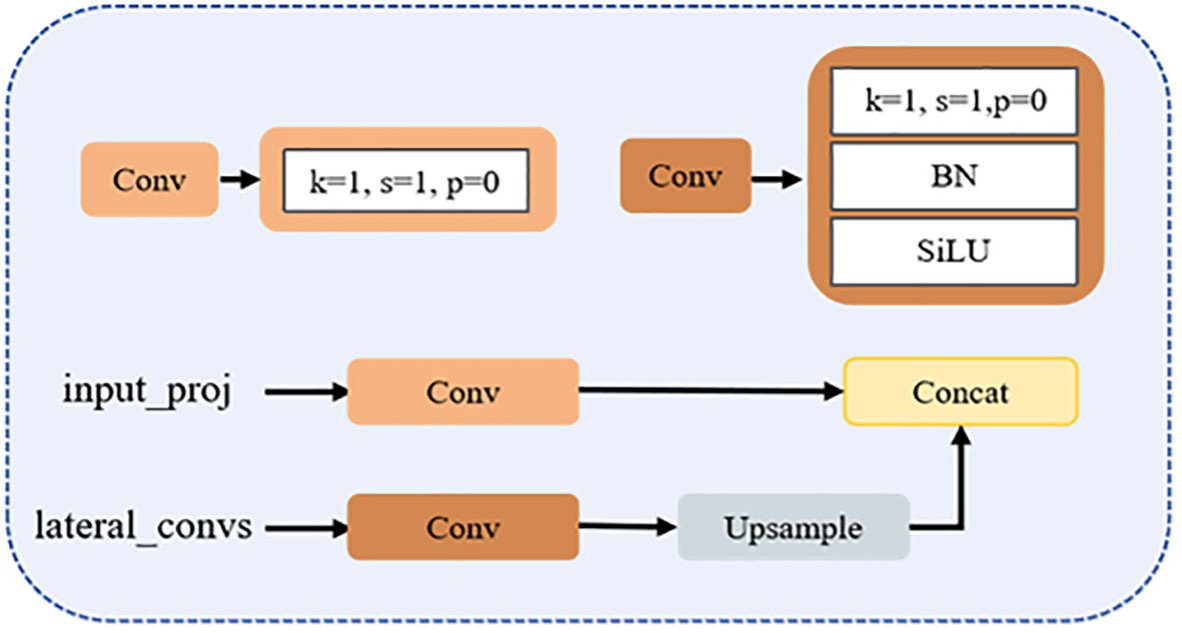
Architecture of the improved cross-channel fusion module (CCFM).

The improved CCFM structure is illustrated in **Figure 7**. The input_proj branch applies a 1×1 convolution to map the output features of the backbone network, achieving channel alignment and feature reorganization. The lateral_convs branch also uses 1×1 convolutions, but omits activation and normalization layers to simplify computation and adjust feature channels. This branch then upsamples the deep features to increase spatial resolution. Once the two feature streams are dimensionally aligned, they are fused through concatenation, effectively integrating high-resolution detail with low-resolution semantic information. In this improved architecture, the RepBlock in **Figure 6** is replaced by an upsampling operation, which boosts both small-object detection capability and feature map resolution, facilitating efficient multi-scale feature fusion.

Compared to the traditional PAN + FPN structure (Guo et al., 2020), the improved neck architecture incorporates four convolution operations for lightweight feature fusion, significantly enhancing the model’s adaptability to multi-scale targets, particularly small objects.

#### 2.2.3 Minimum point distance IoU

The CIoU (Complete Intersection over Union) loss function used in YOLOv10 takes into account the overlap area between the ground truth and predicted boxes, the Euclidean distance between their center points, and the aspect ratio difference. This improves the model’s ability to fit object boundaries. However, in practical applications, when the predicted box and the ground truth box share the same center and aspect ratio, the aspect ratio penalty term in CIoU may degrade to zero—even if there is a significant size mismatch between the two boxes. This reduces the loss function’s sensitivity to boundary dimensions, which can negatively affect model convergence speed and final accuracy.

To address this issue, the MPDIoU (Minimum Point Distance IoU) (Ma and Xu, 2023) loss function is introduced to further enhance the performance of bounding box regression. This method minimizes the Euclidean distance between the top-left and bottom-right corner points of the predicted and ground truth boxes, thereby directly constraining both the size and position of the bounding boxes. The corresponding loss function is expressed as follows:


(6) $$L_{MPDIou}=1-IoU+\frac{d_{1}^{2}}{h^{2}+w^{2}}+\frac{d_{2}^{2}}{h^{2}+w^{2}}$$

(7) $$\begin{array}{c} d_{1}^{2}={(x_{1}^{\mathrm{pred}}+x_{1}^{\mathrm{gt}})}^{2}+{\left(y_{1}^{\mathrm{pred}}+y_{1}^{\mathrm{gt}}\right)}^{2} \end{array}$$

(8) $$\begin{array}{c} d_{2}^{2}={\left(x_{2}^{pred}+x_{2}^{gt}\right)}^{2}+{\left(y_{2}^{pred}+y_{2}^{gt}\right)}^{2} \end{array}$$

In Equations 6–8, ( $x_{1}^{pred}$ , $y_{1}^{pred}$ )and( $x_{2}^{pred}$ , $y_{2}^{pred}$ ) represent the top-left and bottom-right coordinates of the predicted bounding box, respectively. Similarly, ( $x_{1}^{gt}$ , $y_{1}^{gt}$ ) and ( $x_{2}^{gt}$ , $y_{2}^{gt}$ ) denote the top-left and bottom-right coordinates of the ground truth box. www and hhh represent the width and height of the image.

**Figure 8:**
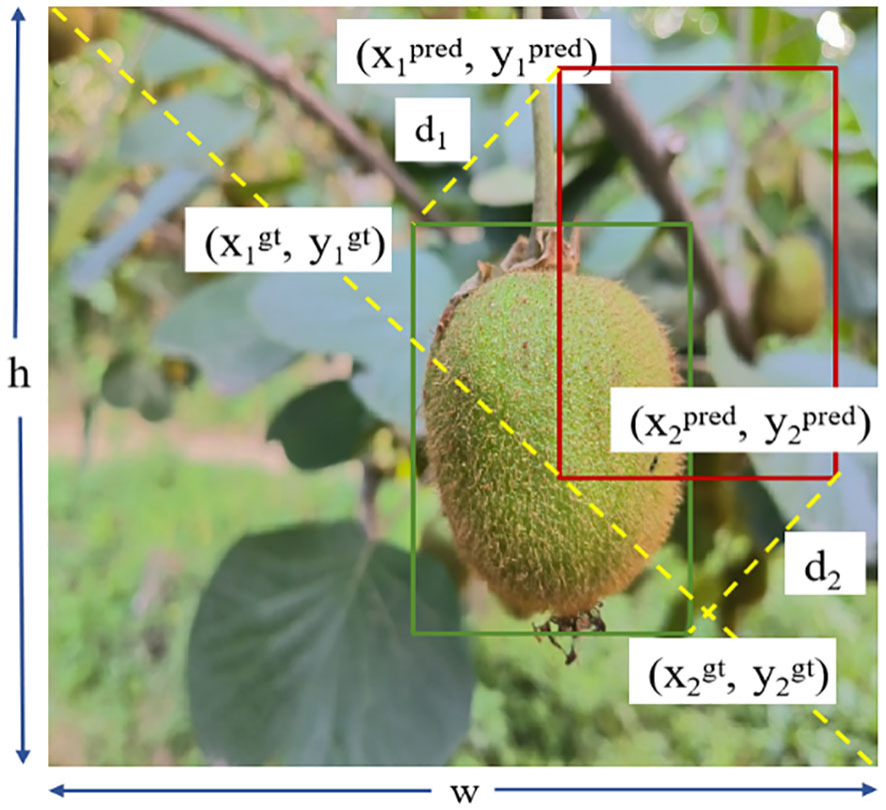
Illustration of MPDIoU calculation process for bounding box regression.

MPDIoU builds upon the optimization of IoU and center point distance by introducing constraints based on the distances between corresponding key points. This enables a more precise measurement of the geometric discrepancy between predicted and ground truth boxes. It effectively addresses the weakness of CIoU, which may fail when aspect ratios are identical but box sizes differ. As a result, MPDIoU enhances both the accuracy of bounding box regression and the model’s convergence speed. An example of MPDIoU metric parameters is illustrated in **Figure 8**.

## 3 Results and analysis

### 3.1 Experimental setup

To ensure the fairness and validity of the experimental results, all models were evaluated on the same standardized experimental platform. The system environment was Ubuntu 20.04, equipped with an Intel^®^ Xeon^®^ Gold 5418Y 12-core processor and an Nvidia GeForce RTX 4090 GPU with 24 GB of memory. The software environment included Python 3.8, PyTorch 1.11, and CUDA 11.3. The training parameters were configured as follows: the initial learning rate was set to 0.01, with a batch size of 16. The SGD optimizer was used, with a weight decay coefficient of 0.0005 and a total of 150 epochs. A warmup strategy with a momentum of 0.937 was adopted to gradually increase the learning rate, using one-dimensional linear interpolation. After the warmup phase, a cosine annealing algorithm was applied to adjust the learning rate throughout the training process. Additionally, the input images were resized to a resolution of 640×640 to ensure consistency in the training data.

### 3.2 Performance evaluation

In the task of kiwifruit detection, algorithm evaluation primarily focuses on two aspects: computational complexity and detection accuracy. Detection accuracy is commonly measured by Precision (P), Recall (R), and Mean Average Precision (mAP). Precision reflects the model’s accuracy in detecting targets, Recall assesses the detection coverage, while mAP, as a comprehensive metric for class-level detection, provides an overall evaluation of the model’s detection performance. Computational complexity is characterized by the number of parameters (Params) and floating point operations per second (FLOPs). A smaller number of parameters indicates a more lightweight model, and lower FLOPs imply reduced computational cost, making the model more suitable for deployment on mobile or edge devices. Meanwhile, the real-time performance of the algorithm is measured by Frames Per Second (FPS); the higher the FPS, the faster the model’s inference speed, and the stronger its real-time capability. These metrics comprehensively reflect the performance of a model from the perspectives of accuracy, computational speed, and complexity, thus providing important references for algorithm optimization and practical deployment. The following is a summary of the formulas for calculating P, R, AP, mAP, Params and GFLOPs.


(9) $$\begin{array}{c} P=\frac{TP}{TP+FP}\times 100\% \end{array}$$

(10) $$\begin{array}{c} R=\frac{TP}{TP+FN}\times 100\% \end{array}$$

(11) $$\begin{array}{c} AP=\int_{0}^{1}P\left(R\right)dR \end{array}$$

(12) $$\begin{array}{c} mAP=\frac{1}{n}\sum_{i-1}^{n}AP_{i}\times 100\% \end{array}$$

(13) $$\begin{array}{c} GFLOP_{S}=\;O\left(\sum_{i=1}^{n}K_{i}^{2}*C_{i}^{2}*C_{i}+\sum_{i=1}^{n}M^{2}*C_{i}\right) \end{array}$$

(14) $$\begin{array}{c} Params=O\left(\sum_{i=1}^{n}M^{2}*K^{2}*C_{i-1}*C_{i}\right) \end{array}$$

In Equation 9, precision P represents the proportion of correctly predicted kiwi instances among all predicted kiwi instances. Here, TP denotes the number of correctly detected kiwis, FP refers to the number of incorrectly detected kiwis, and FN indicates the number of missed (undetected) kiwis.

In Equation 10, recall R represents the proportion of correctly detected kiwi instances out of the total number of actual kiwi instances. In Equation 11, AP denotes the area under the Precision-Recall (P-R) curve. Equation 12 defines mAP as the mean value of AP across all categories.

In this study, since there is only one kiwi category, n = 1. mAP@0.5 indicates the mean average precision when the IoU threshold is set to 0.5. Additionally, in Equations 13 and 14, K represents the kernel size, C is the number of channels, M is the size of the input image, and i denotes the number of iterations.

### 3.3 Comparative results

#### 3.3.1 Comparison of different dataset split ratios

To evaluate the impact of different dataset split ratios on model training, three datasets were constructed with split ratios of 6:2:2, 7:2:1, and 8:1:1 after data annotation. All models were trained using the same parameters. As shown in **Table 1**, the 8:1:1 split achieved the best performance in terms of Recall (85.8%) and mAP@50 (93.6%). Although the 7:2:1 split achieved the highest Precision, its mAP@50 was 92.2%, slightly lower than that of 8:1:1. The 6:2:2 split showed weaker performance in both Recall and mAP@50. Overall, the 8:1:1 split demonstrated the best detection performance, and subsequent model training and validation will be based on this dataset configuration.

**Table 1:** Comparison of model performance across different data split ratios.

Data division	Precision/%	Recall/%	mAP@50/%	mAP@50-95/%
8:1:1	88.4	85.8	93.6	66.4
7:2:1	88.7	85.7	92.2	66.1
6:2:2	87.5	84.4	91.6	65.8

#### 3.3.2 Scaling factor experiment

To achieve the optimal balance between accuracy, inference speed, and resource consumption, we conducted experiments with different scaling factors on the YOLOv10n model. The experimental results are shown in **Table 2**. The original model configuration (depth 0.33, width 0.25) achieved an mAP@50 of 93.6% and the fastest inference speed (1.8 ms), but its parameter count (2.694M) and model size (10.28 MB) are relatively large, making it less suitable for deployment on edge devices.

**Table 2:** Comparison of YOLOv10 model with different scaling factors.

Depth	Width	Precision/%	Recall/%	mAP@50/%	mAP@50-95/%	Params/MB	Model Size/MB	Inference/ms
0.33	0.25	88.4	85.8	93.6	66.4	2.694	10.28	1.8
0.33	0.2	89.5	85.7	93.3	66.4	1.98	7.55	1.8
0.33	0.15	89.6	83.0	92.2	66.4	1.334	5.09	3.4
**0.33**	**0.125**	**87.6**	**82.9**	**92.1**	**65.9**	**0.998**	**3.81**	**2.9**
0.25	0.25	88.4	84.0	93.6	66.4	2.694	10.28	2.5
0.2	0.25	89.0	85.7	93.3	66.7	2.592	9.88	3.7
0.15	0.25	89.0	85.7	93.3	66.7	2.592	9.88	1.6
0.25	0.2	89.5	85.7	93.3	66.4	1.980	7.55	2.3
0.2	0.125	88.1	82.1	91.4	65.1	0.973	3.71	3.6
0.15	0.125	88.1	82.1	91.4	65.1	0.973	3.71	3.3

Through comparison, the configuration with scaling factors (0.33, 0.125) demonstrated excellent performance in terms of mAP@50, parameter count, and model size. Although its inference speed slightly decreased to 2.9 ms, it remains within an acceptable range. This configuration strikes a favorable balance between accuracy and model lightweightness, making it more suitable for edge deployment. Other configurations, such as (0.33, 0.2) and (0.33, 0.15), achieved slightly higher mAP@50, but their overall optimization effect was inferior to that of (0.33, 0.125). The shallower (0.25, 0.2) configuration reached an mAP@50 of 93.3%, but did not significantly reduce the parameter count or model size. Therefore, the (0.33, 0.125) configuration was selected as the optimal choice, offering improved model compactness and inference efficiency while maintaining high accuracy. To aid comparison, the best-performing values in **Tables 2**–**6** are highlighted in bold to indicate the optimal results under each metric.

**Table 3:** Performance comparison of different neck networks.

Neck	Precision/%	Recall/%	mAP@50/%	Params/MB	Model size/MB	GFLOGs
BiFPN	**92.3**	83.1	93.1	0.771	3.02	2.7
Slimneck	83.7	82.7	90.6	1.031	3.81	3.4
CCFM	88.2	**87.0**	**93.3**	**0.586**	**2.36**	**2.32**

**Table 4:** Performance comparison of different loss functions.

Model	Precision/%	Recall/%	mAP@50/%	mAP@50-95/%
Diou	90.1	83.7	92.8	65.8
Eiou	88.5	83.9	92.2	65.7
Giou	90.1	81.7	92.1	65.4
**Mpdiou**	88.3	**86.4**	**93.6**	**66.6**
Shapeiou	90.1	82.1	92	65.3
Siou	**90.4**	82.2	92.3	65.5

**Table 5:** Performance comparison of YOLOv10-kiwi with different detection models on kiwifruit dataset.

Model	Precision/%	Recall/%	mAP@50/%	mAP@50-95/%	Params/M	Model size/MB	GFLOGs	FPS
Faster R-CNN	87.9	**92**	89.9	63.5	42.5	165	250	29
RT-DETR-R18	**90.1**	84.5	**93.8**	**67.6**	19.87	75.82	57.3	38
YOLOv3-tiny	88.7	86	90.9	64.9	12.12	47.4	18.9	54
YOLOv5n	89.3	84.5	93.3	67	2.5	9.55	7.1	76
YOLOv7-tiny	88.5	84.7	91	63.7	6.01	22.95	13.2	71
YOLOv8n	89.6	84.1	93	67	3.01	11.47	8.2	**92.1**
YOLOv9t	88.8	83	93.2	66.6	2.66	10.14	11	83.39
YOLOv10n	88.4	85.8	93.6	66.4	2.7	10.28	6.6	65.1
YOLOv11n	89.9	84.5	93.1	67.1	2.58	10.32	6.4	74.9
Ours	88.3	86.4	93.6	66.6	**0.51**	**2.02**	**2.1**	74

**Table 6:** Ablation study results showing individual contributions of scaling factors, C2fDualHet, CCFM, and MPDIoU.

Model	Scaling factors	C2fDualHet	CCFM	MPDIoU	Precision/%	Recall/%	mAP@50/%	Params/M	Model size/MB	GFLOGs
YOLOv10n					88.4	85.8	93.6	2.7	10.28	5.5
Model 1	✓				87.6	82.9	92.1	0.998	3.81	3.8
Model 2	✓	✓			86.2	83.2	90.9	0.917	3.5	3.3
Model 3	✓		✓		88.2	87	93.3	0.586	2.36	2.32
Model 4	✓			✓	87.8	85.2	92.7	0.998	3.81	3.8
Model 5	✓	✓	✓		**89.6**	83.7	92.8	0.505	2.02	2.1
Ours	✓	✓	✓	✓	88.3	**86.4**	**93.6**	**0.505**	**2.02**	**2.1**

#### 3.3.3 Performance comparison of neck network designs

This study compares the performance of lightweight neck modules—BiFPN (Chen et al., 2021), Slimneck (Li et al., 2022), and CCFM—in object detection tasks. As shown in **Table 3**, CCFM demonstrates a significant advantage in terms of lightweight design, with only 0.586 million parameters, a model size of 2.36 MB, and a computational complexity of 2.32 GFLOPs, outperforming both BiFPN and Slimneck. This makes CCFM particularly suitable for deployment on resource-constrained embedded or mobile devices. Meanwhile, CCFM also delivers excellent detection performance, achieving a recall rate of 87.0%, surpassing BiFPN (83.1%) and Slimneck (82.7%). In summary, CCFM strikes a favorable balance between lightweight design and detection performance, showcasing strong potential for application in resource-limited scenarios.

#### 3.3.4 Comparison of bounding box loss functions

**Figure 9:**
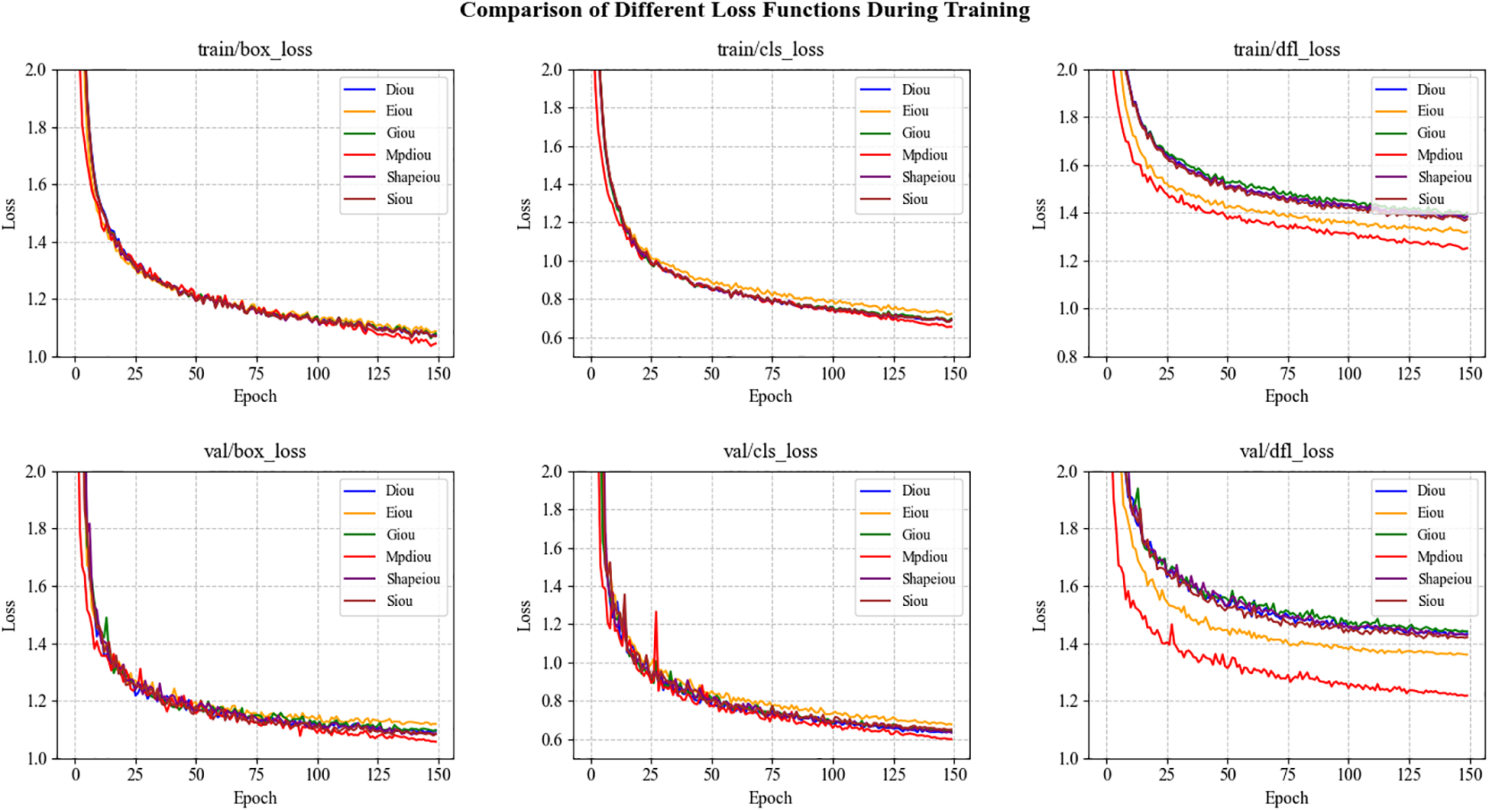
Comparison of training and validation loss curves for various IoU-based loss functions.

We compared six loss functions—DIoU (Zheng et al., 2020), EIoU (Zhang et al., 2022), GIoU (Rezatofighi et al., 2019), CIoU, MPDIoU, and SIoU (Gevorgyan, 2022)—to evaluate their impact on model performance. The experimental results are presented in **Table 4**, and the trends of the loss curves are illustrated in **Figure 9**. According to **Table 4**, MPDIoU demonstrates the most significant performance improvement, with an increase of 0.8% in mAP@50 and 2.7% in Recall, achieving the highest values among all loss functions. Additionally, SIoU yields a 0.8% gain in Precision, while EIoU improves Recall by 0.2%. The remaining loss functions show varying degrees of decline across these key metrics. Further analysis of **Figure 9** reveals that MPDIoU achieves the lowest final values in both val/box_loss and val/cls_loss, with a stable convergence trend, indicating its outstanding robustness in bounding box regression tasks. In contrast, DIoU, EIoU, and GIoU exhibit fluctuations in the loss curves during the later stages of training, suggesting a risk of overfitting. Overall, MPDIoU not only achieves the lowest final loss and fastest convergence on the validation set, but also significantly enhances kiwifruit detection performance—especially in mitigating the impact of low-quality samples such as blurred or occluded instances—demonstrating a well-balanced and superior capability in object detection tasks.

#### 3.3.5 Comparison with other models

**Figure 10:**
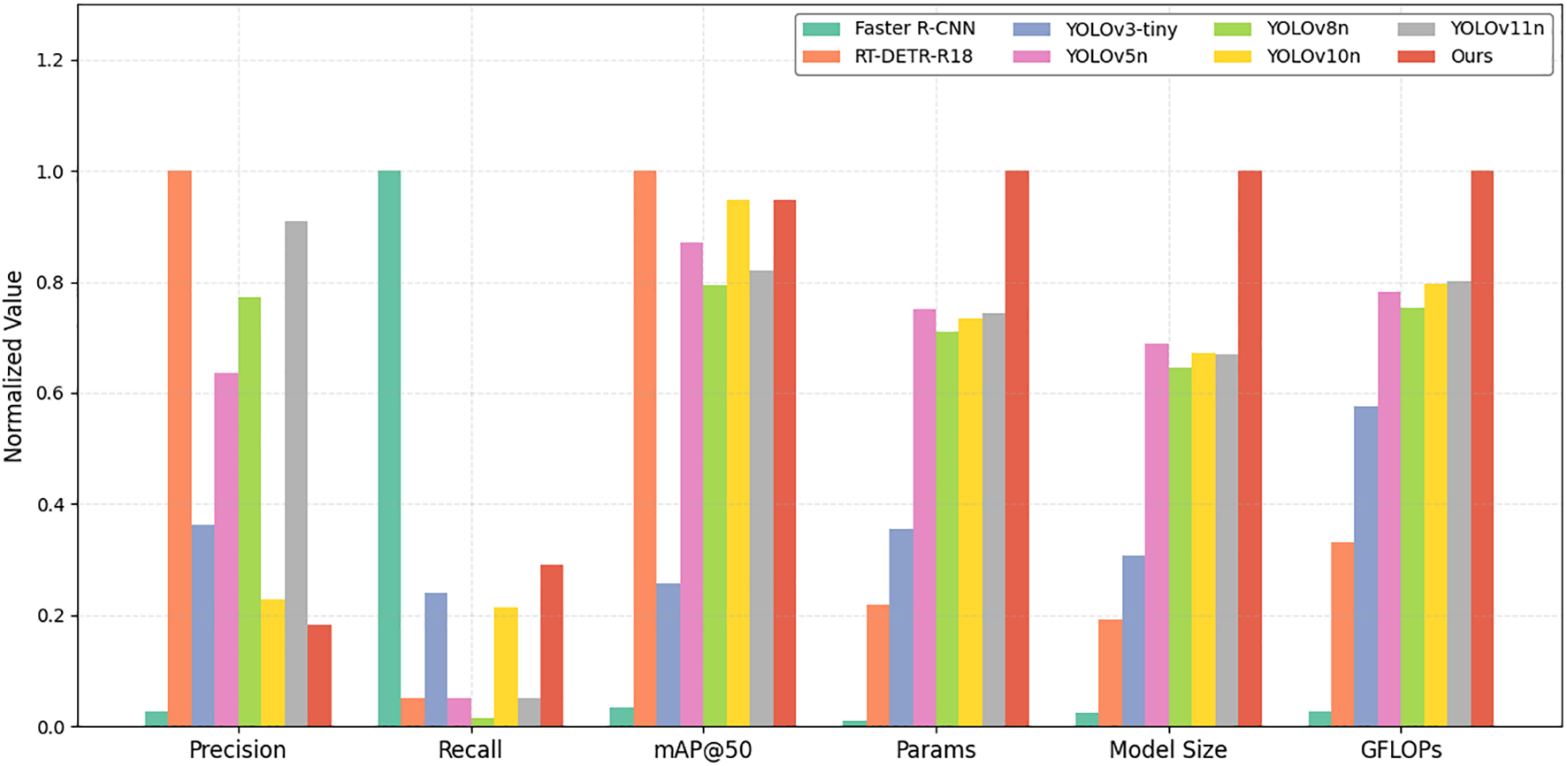
Normalized comparison of accuracy and efficiency among models.

To verify the effectiveness of the proposed model in kiwifruit detection, YOLOv10-Kiwi is compared with nine mainstream object detection algorithms. The results are presented in **Table 5** and **Figure 10**, covering both accuracy and efficiency metrics. In terms of model complexity, YOLOv10-Kiwi contains only 0.51M parameters, which is 98.8%, 97.4%, 95.8%, and 91.5% fewer than those of Faster R-CNN, RT-DETR-R18, YOLOv3-tiny, and YOLOv7-tiny, respectively. Its model size is 2.02 MB, significantly smaller than that of Faster R-CNN and RT-DETR-R18, and 78.8% and 82.4% smaller than YOLOv5n and YOLOv8n. The GFLOPs is only 2.1, which is much lower than most compared models, including YOLOv8n (8.2) and YOLOv10n (6.6). Despite its compact structure, YOLOv10-Kiwi achieves competitive detection performance. It reaches an mAP@50 of 93.6%, comparable to RT-DETR-R18 and YOLOv10n, and 4.1% and 2.7% higher than Faster R-CNN and YOLOv3-tiny, respectively. The recall is 86.4%, second only to Faster R-CNN (92%). The inference speed reaches 74 FPS, close to YOLOv5n (76) and YOLOv7-tiny (71), demonstrating real-time capability.

**Figure 10** provides a visual comparison of six normalized metrics, where detection-related indicators (precision, recall, mAP@50) are positively normalized and resource-related metrics (parameters, model size, GFLOPs) are reverse-normalized. YOLOv10-Kiwi exhibits a well-balanced profile, combining high detection accuracy with superior efficiency. These results highlight its strong potential for deployment on resource-constrained edge devices in agricultural scenarios.

### 3.4 Ablation experiments

**Figure 11:**
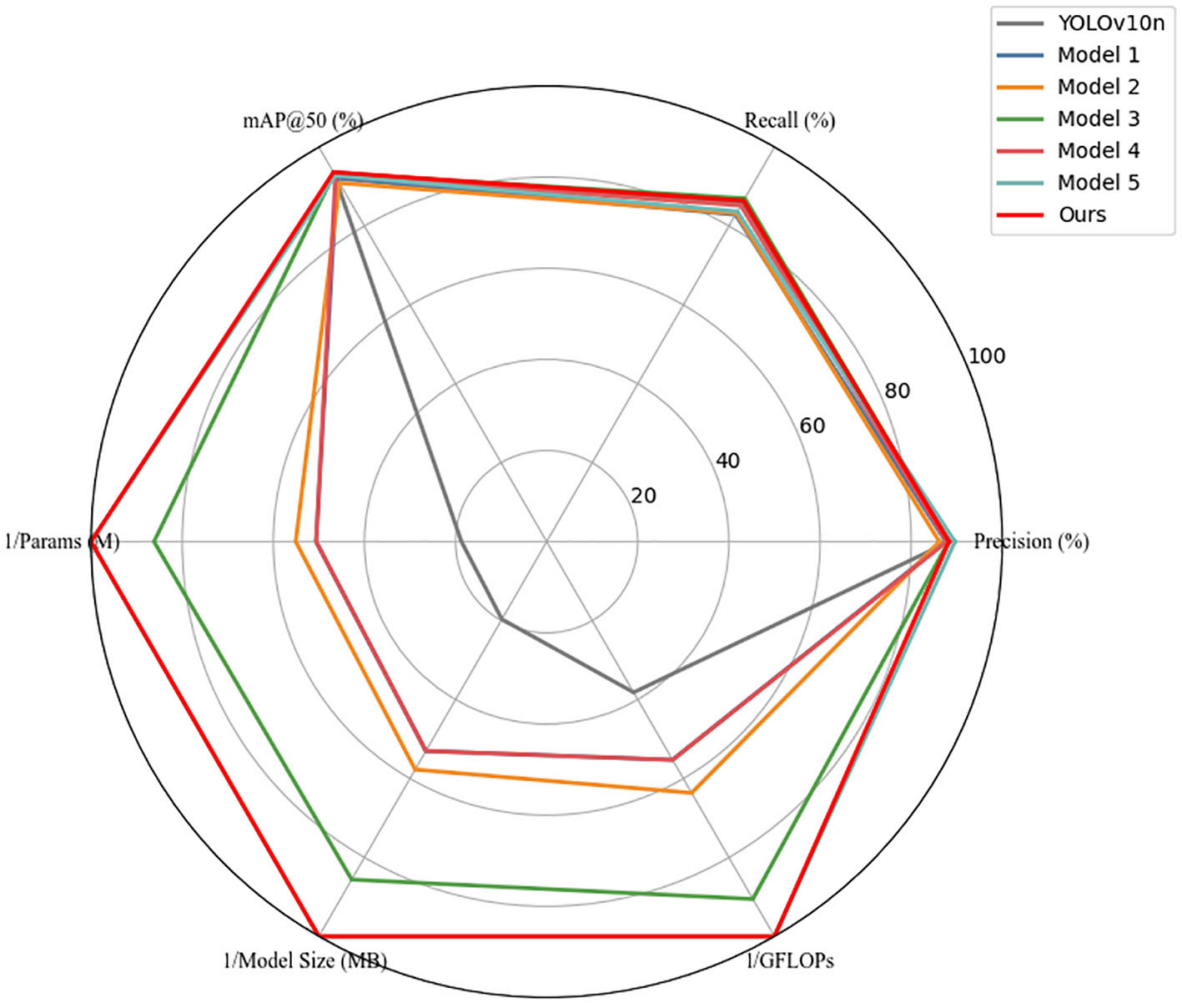
Radar chart comparing accuracy and complexity of ablation models.

To validate the effectiveness of the proposed lightweight algorithm in kiwifruit object detection tasks, we conducted a series of systematic ablation experiments. The results are shown in **Table 6** and **Figure 11**.

First, by adjusting the scaling factors of the YOLOv10n network, we obtained Model 1, which significantly reduced model complexity—parameters decreased from 2.7M to 0.998M (a reduction of 63.04%) and GFLOPs from 5.5 to 3.8 (a reduction of 30.91%)—demonstrating that scaling-based compression can effectively shrink the model footprint. However, detection performance declined, with Recall dropping by 2.9% and mAP@50 by 1.5%, indicating a degradation in representational capacity due to downscaled feature extraction. To address this, Model 2 introduced the C2fDualHet module as a lightweight replacement for the original C2f block in the backbone. The module was designed to further reduce computational overhead while enhancing the network’s ability to capture local and global features. Compared to Model 1, the parameter count and GFLOPs were further reduced by 8.12% and 13.16%, respectively—confirming that the compression objective was further achieved. However, detection accuracy remained relatively unchanged, and mAP@50 slightly decreased by 0.7%, suggesting that while C2fDualHet successfully reduces computation, its standalone impact on detection precision is limited without enhanced feature fusion. Model 3, which added the Cross-Channel Fusion Module (CCFM) to Model 1, significantly improved performance. Recall increased from 82.9% to 87.0%, and mAP@50 rose from 92.1% to 93.3%, with Precision reaching 88.2%. This confirms CCFM’s role in strengthening feature integration across scales and channels, compensating for information loss from earlier compression. Model 4 evaluated the impact of using MPDIoU loss in isolation (on top of Model 1). Compared with Model 1, Recall improved markedly from 82.9% to 85.2%, while mAP@50 increased to 92.7%, demonstrating that MPDIoU can significantly improve localization performance without structural changes. Model 5 combined both C2fDualHet and CCFM, resulting in the highest Precision (89.6%) among all sub-models and achieving strong efficiency with only 0.505M parameters and 2.1 GFLOPs, highlighting the synergistic benefits of both modules. Finally, our full model (Ours) integrated all proposed components, including MPDIoU loss. It achieved the best balance overall, with mAP@50 of 93.6%, Recall of 86.4%, and a compact model size of 2.02 MB. These results confirm that the full architecture of YOLOv10-Kiwi effectively balances detection accuracy and lightweight design.

To intuitively illustrate the performance trade-offs, **Figure 11** presents a radar chart with normalized values (0–1), where computational metrics were inversely scaled. The progressive expansion of the radar area clearly visualizes how each component contributes to the overall model performance, with YOLOv10-Kiwi (Model Ours) demonstrating the best balance across all metrics.

### 3.5 Model detection effect analysis

**Figure 12:**
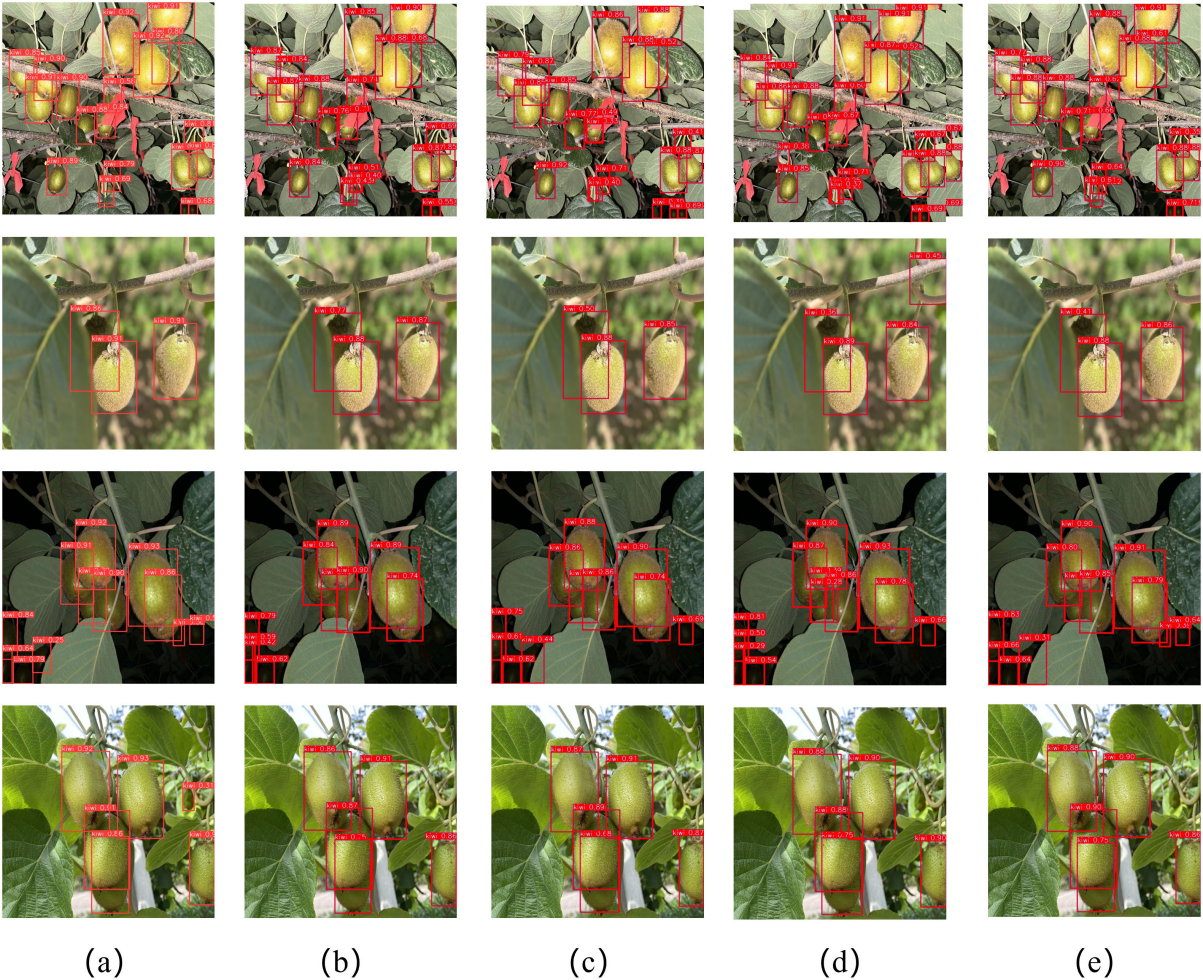
Detection results of different models. **(a)** RT-DETR-R18; **(b)** YOLOv3-tiny; **(c)** YOLOv8n; **(d)** YOLOv10n; **(e)** YOLOv10-Kiwi.

To evaluate the performance of YOLOv10-Kiwi in the kiwifruit detection task, we present the detection results of RT-DETR-R18, YOLOv3-tiny, YOLOv8n, YOLOv10n, and YOLOv10-Kiwi in **Figure 12**. YOLOv10-Kiwi is capable of accurately identifying kiwifruit and maintains high detection precision even in complex backgrounds. Although occlusion from branches and leaves or lighting variations may cause occasional false detections or missed detections, the overall impact is minimal, indicating that YOLOv10-Kiwi can effectively meet the requirements of kiwifruit detection. In comparison, RT-DETR-R18 demonstrates limited detection accuracy and is prone to false detections under complex backgrounds; YOLOv3-tiny shows improvements in reducing false positives but still suffers from missed detections; YOLOv8n and YOLOv10n strike a balance between accuracy and real-time performance, yet exhibit deviations in detecting partially occluded targets. Notably, as a lightweight model, YOLOv10-Kiwi significantly reduces parameter count and computational complexity while maintaining detection accuracy comparable to YOLOv10n, making it more suitable for deployment on resource-constrained edge devices. YOLOv10-Kiwi effectively reduces the missed detection rate while ensuring high confidence, demonstrating outstanding detection performance.

### 3.6 Model visualization analysis

**Figure 13:**
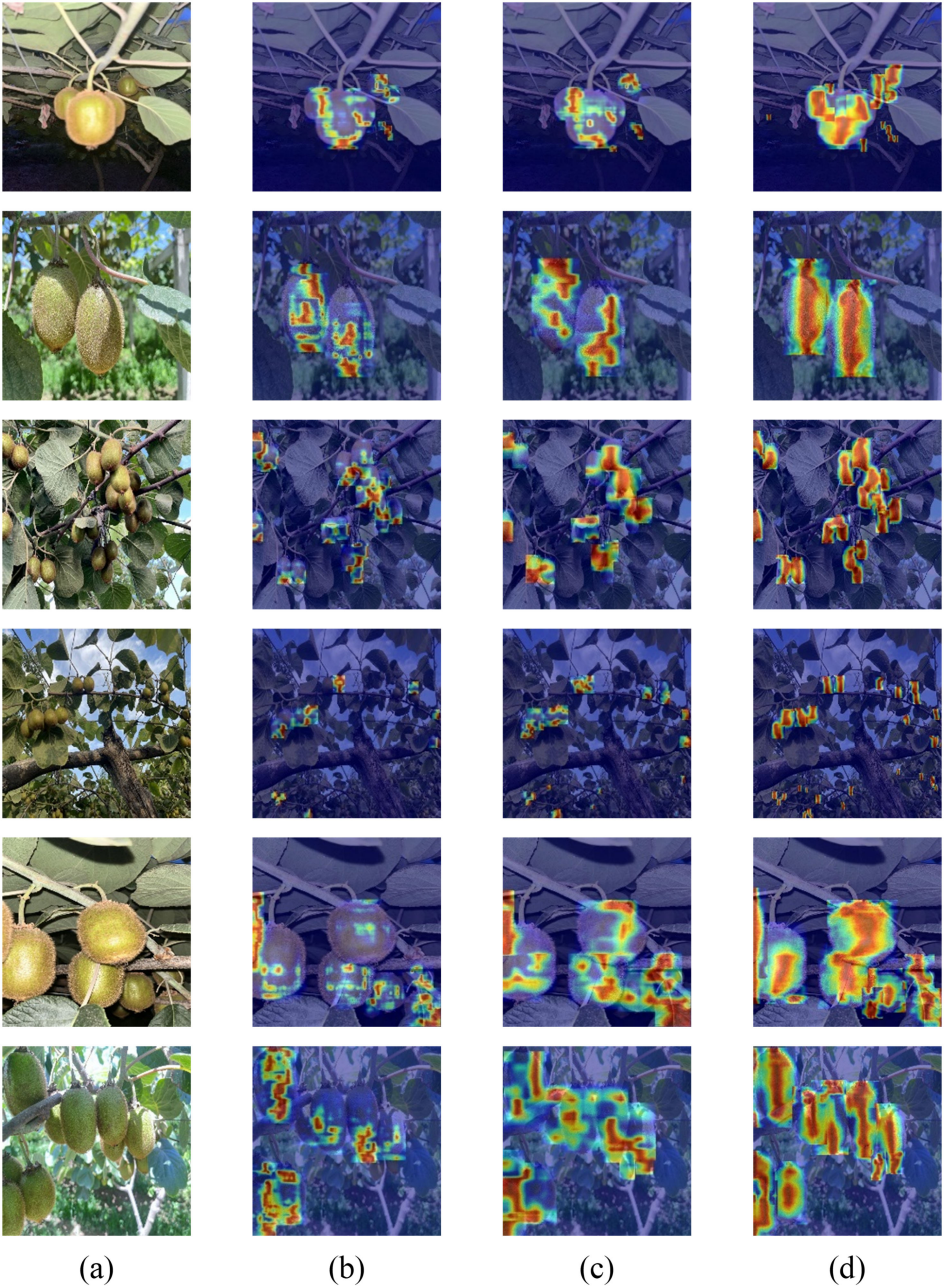
Heat map for different models. **(a)** original images; **(b)** YOLOv3-tiny; **(c)** YOLOv8n; **(d)** YOLOv10-Kiwi.

To further investigate the feature extraction capabilities of the model in kiwifruit detection, this study extracts features from multiple convolutional layers of different models and utilizes the Grad-CAM method (Selvaraju et al., 2017)to generate heatmaps for visual analysis, as shown in **Figure 13**. In these heatmaps, the regions of higher attention toward the kiwifruit targets are represented by deeper red areas, while lighter areas reflect lower attention. Experimental results indicate that YOLOv10-Kiwi demonstrates excellent performance in multi-layer feature extraction. Compared to YOLOv3-tiny and YOLOv8n, its heatmaps exhibit more focused and precise responses in the target regions, particularly in the identification of kiwifruit under complex backgrounds. This suggests that YOLOv10-Kiwi holds significant advantages in the hierarchical structure and accuracy of feature extraction, making it more suitable for the task of kiwifruit detection.

## 4 Conclusion

In this study, we proposed YOLOv10-Kiwi, a lightweight and high-performance model specifically designed for kiwifruit detection in complex orchard environments. By integrating the C2fDualHet module, CCFM neck, and MPDIoU loss into the YOLOv10n framework, the model achieves an effective trade-off between accuracy and computational efficiency. Extensive experiments demonstrated that YOLOv10-Kiwi achieves an mAP@50 of 93.6%, recall of 86.4%, and precision of 88.3%, while reducing parameters to 0.51M, model size to 2.02 MB, and GFLOPs to 2.1. The real-time inference speed reaches 74 FPS, making it highly suitable for deployment on edge devices in agricultural scenarios. The proposed model exhibits robust detection under various conditions, such as occlusion, lighting variations, and complex backgrounds. It is also much more lightweight than comparable state-of-the-art detectors, enabling its practical use in resource-constrained environments.

In future work, we plan to expand the dataset across multiple seasons and kiwifruit varieties, and validate the model’s generalizability under varying orchard geometries. Moreover, we aim to integrate YOLOv10-Kiwi into robotic picking platforms to further explore its applicability in real-world agricultural automation tasks.

## Data Availability

The original contributions presented in the study are included in the article/supplementary material. Further inquiries can be directed to the corresponding author.
